# National evaluation of rapidly growing mycobacteria outbreaks in Brazil

**DOI:** 10.1186/1753-6561-5-S6-P98

**Published:** 2011-06-29

**Authors:** MC Padoveze, G Madalosso, DJ Hadad, E Bravo, EC Silva, HM Borba, J Sallas, JLM Sampaio, JF Ribeiro, MDS Santos, RS Duarte, SM Gomes, SC Leão, VR Brilhante

**Affiliations:** 1 Work Group for Outbreak Investigation, Agência Nacional de Vigilância Sanitária and Health Surveillance Secretariat from Brazilian Ministry of Health, Brasília, Brazil

## Introduction / objectives

This study describes the epidemiologic characteristics of HAI outbreaks caused by RGM in Brazil.

## Methods

A retrospective study was carried out by consulting the Agência Nacional de Vigilância Sanitária (ANVISA), Brazil, data system from January 1999 to December 2009. Additional data from private and public laboratories were also analyzed, including genotyping of a large group of RGM by means of Pulsed-field Gel Electrophoresis (PFGE). Cases were defined as confirmed, probable or suspect according to a previously established definition.

## Results

2,520 RGM infections were reported to ANVISA from 23 States during the period studied. Confirmed cases were caused by *M. abscessus* (31.3%, n=265), *M abscessus* subsp. *bolletti* (30.4%, n=257), *M. fortuitum* (13.8%, n=117), and other species contributed to 4.2% of the cases (n=34). Cases were mainly associated with videoscopy procedures (n=1,722), mammaplasty (n=210) or nonsurgical invasive procedures (n=141). PFGE fingerprints showed evidence of a nationwide spread of a single strain of *M abscessus subsp. bolletti*, mainly in cases involving video-assisted procedures, thus suggesting a common source. Due to the lack of relevant information we could not confirm the hypothesis generated by the descriptive study. Measures to control outbreaks and prevent new cases were adopted by ANVISA, including a sanitary audit, guideline publication and training programs.

## Conclusion

Outbreaks involving RGM occurred with different epidemiologic features in Brazil during the period studied. Further studies are necessary to identify the factors implicated in the persistence and dissemination of a single clone throughout the country.

## Disclosure of interest

None declared.

